# Identification of the metabolic alterations associated with the multidrug resistant phenotype in cancer and their intercellular transfer mediated by extracellular vesicles

**DOI:** 10.1038/srep44541

**Published:** 2017-03-17

**Authors:** Vanessa Lopes-Rodrigues, Alessio Di Luca, Justyna Mleczko, Paula Meleady, Michael Henry, Milica Pesic, Diana Cabrera, Sebastiaan van Liempd, Raquel T. Lima, Robert O’Connor, Juan M. Falcon-Perez, M. Helena Vasconcelos

**Affiliations:** 1 i3S - Instituto de Investigação e Inovação em Saúde, Universidade do Porto, Portugal; 2 Cancer Drug Resistance Group, IPATIMUP - Institute of Molecular Pathology and Immunology of the University of Porto, 4200-465 Porto, Portugal; 3 ICBAS-UP - Institute of Biomedical Sciences Abel Salazar, University of Porto, 4099-003 Porto, Portugal; 4 NICB - National Institute for Cellular Biotechnology, Dublin City University, Dublin 9, Ireland; 5 Exosomes Laboratory & Metabolomics platform, CIC bioGUNE, CIBERehd, Derio, Spain; 6 Institute for Biological Research “Sinisa Stankovic”, University of Belgrade, Despota Stefana 142, 11060 Belgrade, Serbia; 7 Department of Pathology - FMUP - Faculty of Medicine of the University of Porto, Porto, Portugal, Alameda Prof. Hernâni Monteiro, 4200-319 Porto, Portugal; 8 IKERBASQUE, Basque Foundation for Science, 48011 Bilbao, Spain; 9 Department of Biological Sciences, FFUP - Faculty of Pharmacy, University of Porto, 4050-313 Porto, Portugal

## Abstract

Multidrug resistance (MDR) is a serious obstacle to efficient cancer treatment. Overexpression of P-glycoprotein (P-gp) plays a significant role in MDR. Recent studies proved that targeting cellular metabolism could sensitize MDR cells. In addition, metabolic alterations could affect the extracellular vesicles (EVs) cargo and release. This study aimed to: i) identify metabolic alterations in P-gp overexpressing cells that could be involved in the development of MDR and, ii) identify a potential role for the EVs in the acquisition of the MDR. Two different pairs of MDR and their drug-sensitive counterpart cancer cell lines were used. Our results showed that MDR (P-gp overexpressing) cells have a different metabolic profile from their drug-sensitive counterparts, demonstrating decreases in the pentose phosphate pathway and oxidative phosphorylation rate; increases in glutathione metabolism and glycolysis; and alterations in the methionine/S-adenosylmethionine pathway. Remarkably, EVs from MDR cells were capable of stimulating a metabolic switch in the drug-sensitive cancer cells, towards a MDR phenotype. In conclusion, obtained results contribute to the growing knowledge about metabolic alterations in MDR cells and the role of EVs in the intercellular transfer of MDR. The specific metabolic alterations identified in this study may be further developed as targets for overcoming MDR.

The development of multidrug resistance (MDR) in cancer is a serious impediment to treatment success. MDR is defined as a phenotype of the cells resistant to multiple structurally and functionally different drugs. Such resistance is multifactorial and may be due to various mechanisms^1^,^2^. There are several important mechanisms involved in MDR whose identification has generated valuable information on how to circumvent MDR and improve chemotherapy treatment.

One of the most important known mechanism is the overexpression of ATP-binding cassette (ABC) transporters, commonly known as drug efflux pumps, such as P-glycoprotein (P-gp)^2^, which is frequently overexpressed in cancer^3^. P-gp transports drug-substrates across the cell membrane, thus decreasing their intracellular concentrations to sub-lethal^4^.

Several studies pointed to a relation between MDR and alterations in cellular metabolism: (i) upregulation of hypoxia-induced factor 1 (HIF-1) was shown to be associated with chemoresistance^5^; (ii) leukemia models with higher glycolytic rates were resistant to glucocorticoids^6^; (iii) modulation of cellular metabolic pathways was demonstrated to contribute to acquired resistance in multiple myeloma cells^7^; (iv) glycolytic pyruvate was capable of regulating P-gp expression in multicellular tumor spheroids^8^; and (v) hypoxia was shown to induceMDR and glycolysis in an orthotopic MDR tumor model in nude mice^9^. Ultimatelly, these studies may contribute to understanding how MDR could be circumvented by application of specific metabolic modulators and inhibitors. Therefore, it is important to identify metabolic alterations in MDR cancer cells, which could lead to the identification of new metabolic molecular targets to circumvent MDR in cancer.

The formation of Extracellular vesicles (EVs) and their release have been implicated in pathological processes such as cancer^10^,^11^,^12^ and shown to be relevant for the intercellular transfer of a drug-resistant phenotype^12^,^13^,^14^. Indeed, drug-sensitive cancer cells can become drug-resistant following intracellular incorporation of EVs shed by drug-resistant cancer cells^13^,^14^,^15^,^16^. We have previously shown that the EVs population shed by MDR cells is different from the one shed by drug-sensitive counterpart cells, thus suggesting that MDR cells produce more microvesicles and less exosomes than their drug-sensitive counterpart cells^17^. In addition, several studies have stated that metabolic alterations in cancer cells could induce alterations in the EVs’ cargo and its release^18^,^19^,^20^. So far, it is unclear if these metabolic alterations are caused by or could be responsible for the MDR phenotype.

Here we provide evidence that MDR cancer cell lines (overexpressing P-gp) acquired a different metabolic profile from their drug-sensitive counterpart cells and that the EVs released by MDR cells caused a metabolic switch towards the MDR phenotype in the recipient cells.

## Results

### Protein profiling and bioinformatics analysis of MDR and drug-sensitive counterpart cell lines identified differentially expressed proteins (DEPs)

For protein profiling, each of the four biological replicates of each condition was run by LC–MS. The data was transferred to *Progenesis QI* for proteomics to compare drug-sensitive cancer cells (K562 and NCI-H460) with their MDR counterparts (K562Dox and NCI-H460/R). Individual comparisons were carried out for each pair of cell lines: K562 *versus* K562Dox and NCI-H460 *versus* NCI-H460/R. Following Progenesis LC–MS analysis, peptide features with ANOVA < 0.05 and 1+, 2+ and 3+ charge states were subjected to MASCOT database searching. The MASCOT mgf files were then resubmitted to the Progenesis software to yield a list of identified proteins. These lists were further interrogated to exclude proteins with less than 2 peptides matched, a fold change <1.5 and not statistically significant.

A total of 91 significant (*p* < 0.05) differentially expressed proteins (DEPs) were identified when comparing the K562 *vs*. K562Dox cells and 67 significant DEPs (*p* < 0.05) were identified when comparing the NCI-H460 *vs*. NCI-H460/R cells. A full list of the DEPs identified for each cell line is available in the Supplementary Information (Tables S1 and S2).

### Gene Ontology (GO) bioinformatics analysis indicated that the greatest difference between MDR cells and their drug-sensitive counterparts occurred in metabolic processes

**Figure 1:**
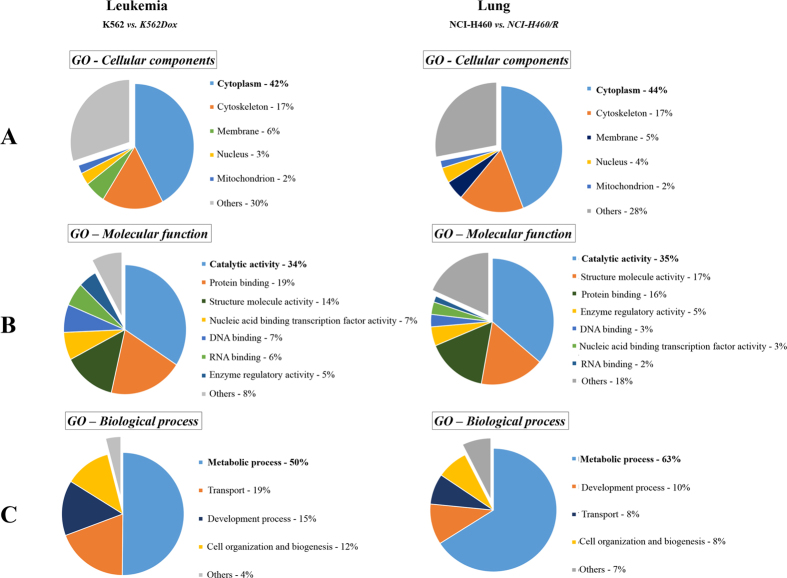
GO analysis of all the differentially expressed proteins (DEPs) identified with the *Progenesis QI* software in both pairs of counterpart drug-sensitive and MDR cancer cell lines: K562 *versus* K562Dox and NCI-H460 *versus* NCI-H460/R. (**A**) GO - Cellular component analysis of the identified proteins; (**B**) GO - Molecular functional analysis of the identified proteins; and (**C**) GO - Biological process analysis of the identified proteins.

Using the *Database for Annotation, Visualization and Integrated Discovery (DAVID*) network analysis tool, the molecular functions/localizations of the DEPs data sets were analyzed according to GO functional annotations and categories. GO analysis of cellular components, molecular functions and biological processes were performed on the 91 DEPs (K562 *vs*. K562Dox) and 67 DEPs (NCI-H460 *vs*. NCI-H460/R) identified with the *Progenesis* software. Pie diagrams represent the GO analysis of the identified DEPs (Fig. 1). The GO analysis revealed that most of the DEPs (for both cancer cell models) have cytoplasmic origin (42% in K562 *vs*. K562Dox and 44% in NCI-H460 *vs*. NCI-H460/R), cytoskeleton origin (17% for both models) and membrane localization (6% in K562 *vs*. K562Dox and 5% in NCI-H460 *vs*. NCI-H460/R) (Fig. 1A). The functional classification of these proteins from both cancer cell models implied that they are mostly involved in catalytic activities, structure molecule activities and protein binding activities (Fig. 1B). Noteworthy, analysis of the biological processes indicated that the greatest difference between the MDR cells and their drug-sensitive counterparts occurred in metabolic processes (50% in K562 *vs*. K562Dox and 63% in NCI-H460 *vs*. NCI-H460/R) (Fig. 1C). The remaining different biological processes were associated with transport, development processes and cell organization and biogenesis.

### KEGG pathway enrichment analysis indicated that the most significant active pathways enriched in the MDR cells were involved in metabolic processes

Following the identification of the DEPs, the most significant active pathways were analyzed using KEGG pathway enrichment analysis. Results indicated that the most significantly active pathways enriched in the MDR cells were those involved in metabolic processes. The glutathione metabolism (GSH), pentose phosphate pathway (PPP) and glycolysis were found to be the most enriched pathways identified in both cancer cell models (Table 1). In the chronic myeloid leukemia model (K562Dox) the following 4 DEPs were found to be involved in the GSH metabolism pathway (G6PD, PRDX2, IDH1 and 6PGD), 3 DEPs in the PPP pathway (G6PD, ALDOC and 6PGD) and 2 DEPs in the glycolysis pathway (ALDOC and PKM2). In the non-small cell lung cancer model the following 5 DEPs were found to be involved in the GSH metabolism pathway (G6PD, PRDX2, IDH1, MGST1 and 6PGD), 4 DEPs in the PPP pathway (G6PD, TALDO1, TKT and 6PGD) and 1 DEP involved in the glycolysis pathway (ALDH3A1) (Table 1).

### Other DEPs involved in MDR and metabolic processes were identified by bioinformatics analysis

In addition to the DEPs referred above (Table 1) other DEPs also involved in metabolic process and MDR were analyzed in terms of normalized abundance between MDR and drug-sensitive counterpart cells (for both cancer cell models) (Table 2).

The DEP with the highest fold change was P-gp, which was upregulated in both MDR cells with a fold change of 18.8 for K562Dox *vs*. K562 and 64.5 for NCI-H460/R *vs*. NCI-H460. In terms of metabolic processes, most of the DEPs involved in the PPP pathway were downregulated in the two MDR models (ALDOC, G6PD, 6PGD and TKT) and only TALDO1 was upregulated in the NCI-H460/R cells (Table 2). Regarding GSH metabolism, the enzymes responsible for the NADP^+^ reduction to NADPH (G6PD, 6PGD and IDH1) were downregulated in the MDR cells but MGST1 was upregulated in NCI-H460/R cells. Regarding glycolysis, ALDOC was downregulated in MDR cells but the enzyme responsible for the pyruvate production (PKM2) was upregulated. Additionally, DEPs involved in the TCA cycle were also identified (ACLY and ACO2). ACLY was upregulated whereas ACO2 was downregulated in the MDR cancer cell models (Table 2).

Interestingly HYOU1 and NDRG1 (both involved in cellular response to hypoxia) and MTHFD1 (an enzyme involved in the methionine pathway) were upregulated in the MDR cells (Table 2).

### Results obtained by quantitative label-free proteomic approach were validated by Western blot

**Figure 2:**
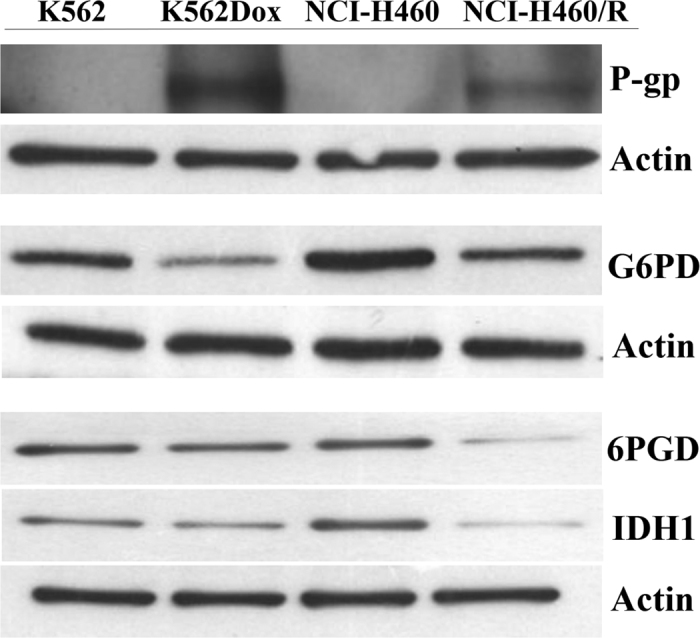
Validation by Western blot analysis of some DEPs initially identified by quantitative proteomics. Representative blots were chosen from three independent experiments. Actin was used as a loading control.

The expression of P-gp, G6PD, 6PGD and IDH1 were further validated by Western blot. We focused the validation on DEPs involved in MDR, glutathione metabolism and pentose phosphate pathway. Consistent with the results obtained with the proteomic analysis, P-gp was found upregulated while G6PD, 6PGD and IDH1 were downregulated in the MDR cells (K562Dox and NCI-H460/R) when compared with their drug-sensitive counterpart cells (K562 and NCI-H460) (Fig. 2).

### MDR cells presented higher levels of reduced glutathione (GSH) and lower levels of reactive oxygen species (ROS)

Since most of the proteins that had been identified as DEPs in MDR cells were related to metabolic processes such as GSH metabolism and PPP, the GSH (one of the main detoxification agents in the cell) and ROS levels were analyzed in both tumor models.

**Figure 3:**
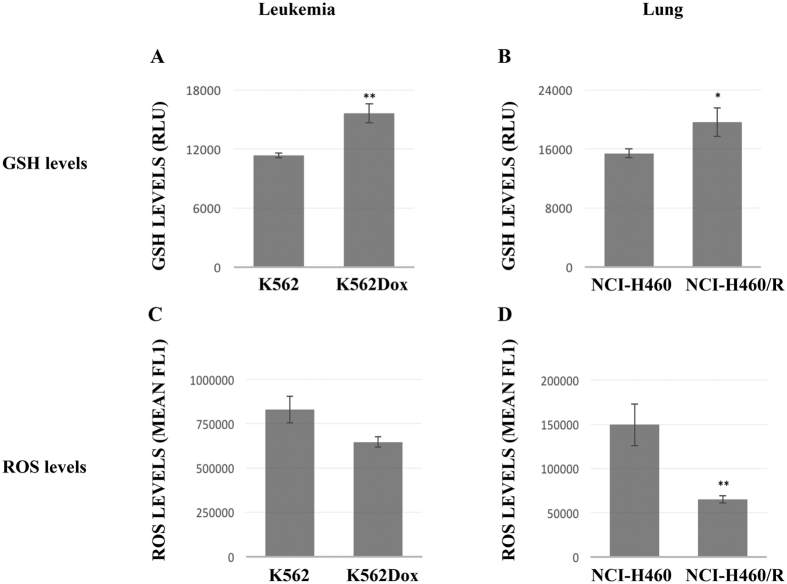
Comparison of GSH and ROS levels between drug-sensitive and MDR cancer cell lines. (**A**) GSH levels in K562 and K562Dox cells. (**B**) GSH levels in NCI-H460 and NCI-H460/R cells. GSH levels are represented as Relative Luminescence Units (RLU). (**C**) ROS levels in K562 and K562Dox cells. (**D**) ROS levels in H460 and NCI-H460/R cells. The ROS levels are represented as mean fluorescence. Staurosporine was used as a positive control. Results are the mean ± SEM of 3 independent experiments. *p ≤ 0.05 and **p ≤ 0.01.

MDR cells (K562Dox and NCI-H460/R) presented significantly higher levels of GSH when compared to their drug-sensitive counterpart cells (K562 and NCI-H460) (Fig. 3A,B). In agreement with this higher capacity provided by the higher levels of GSH to respond to oxidative stress, MDR cells presented lower levels of ROS when compared to their drug-sensitive counterpart cells ( 3C,D).

### Changes in methionine/S-adenosylmethionine pathway were identified in the MDR cancer cells

As we found GSH significantly increased in MDR cells, we were also interested in studying the metabolic pathways involved in the biosynthesis of GSH. The main cellular pathway that generates precursor for the synthesis of GSH corresponds to the methionine/S-adenosylmethionine (SAMe) pathway. By UPLC-MS metabolomics^21^ we have quantified metabolites belonging to this pathway in the MDR and their drug-sensitive counterpart cells (Table 3 and Table S3). Methionine was upregulated in both MDR cancer cells. On the contrary, S-adenosylhomocysteine (SAH) and spermidine were downregulated in both MDR cancer cell lines (K562Dox and NCI-H460/R) (Table 3). In addition, no alterations in S-adenosylmethionine (SAMe) were found in both cancer cell models (Table 3). Some other metabolites were found altered (such as (MTA, dcSAMe, betaine, choline and serine) but in different manners between the two cancer models.

Furthermore, the methylation index (SAMe/SAH), which represents the methylation capacity of the cells, was higher in MDR cells. In the chronic myeloid leukemia model, K562 cells had a methylation index of 6.97, whereas the methylation index for K562Dox cells was 12.05. In the non-small cell lung cancer model, NCI-H460 cells had a methylation index of 2.43 whereas the methylation index for NCI-H460/R cells was 9.79.

### MDR cells (NCI-H460/R) showed an increase in the non-glycolytic acidification, glycolysis, glycolytic capacity and glycolytic reserve

In order to gain new insights into the metabolic phenotype of MDR cells, the extracellular acidification rate (ECAR), which reflects the rate of glycolysis, and the oxygen consumption rate (OCR), which reflects the rate of oxidative phosphorylation (OXPHOS), were measured in both MDR and sensitive cancer cells. Measurements were performed in both basal cellular state and after treatments with compounds capable of modulating glycolysis and OXPHOS.

**Figure 4:**
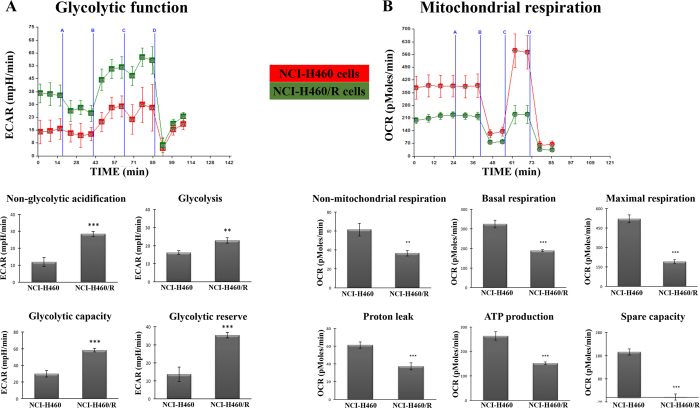
Global metabolic differences between the NCI-H460 and NCI-H460/R counterpart cell lines. Cells were metabolically profiled using *Seahorse XF*-*24 Analyser*. (**A**) Representative results of a glycolysis stress test, which measures extracellular acidification rate (ECAR) following addition of glucose-free media (blue line A), glucose (blue line B), oligomycin (blue line C) and 2-deoxyglucose (blue line D). (**B**) Representative results of a mitochondrial stress test, measuring the oxygen consumption rate (OCR) in glucose-containing media, following sequential addition of media (blue line A), oligomycin (blue line B), FCCP (blue line C) and rotenone (blue line D). Results are the mean ± SEM from three independent experiments, with four to eight replicates per experiment. *p ≤ *0.05*; **p ≤ *0.01*; ***p ≤ 0.001 of NCI- H460 *vs*. NCI-H460/R cells.

To analyze the glycolytic function, a glycolysis stress test was performed. The ECAR was measured in cells treated sequentially with: their glucose-free assay media as a control (Fig. 4A, blue line A); then with glucose to allow the cells to enter into glycolysis (Fig. 4A, blue line B); next with olygomycin (an ATP coupler) in order to inhibit ATP synthesis by blocking the proton channel and shifting the energy production to glycolysis (Fig. 4A, blue line C); and finally with 2-deoxyglucose (2-DG) which is a glucose analog that inhibits glycolysis through competitive binding to glucose hexokinase (Fig. 4A, blue line D). The resulting data allowed calculating the following parameters: Non-glycolytic acidification (= last rate measurement prior to glucose injection); Glycolysis (= maximum rate measurement before oligomycin injection – last rate measurement before glucose injection); Glycolytic capacity (= maximum rate measurement after oligomycin injection – last rate measurement before glucose injection); Glycolytic reserve (= glycolytic capacity – glycolysis).

In terms of glycolytic function, the MDR cells (NCI-H460/R) showed a statistically significant increase in the non-glycolytic acidification, glycolysis, glycolytic capacity and glycolytic reserve, when compared to their drug sensitive counterpart cells (NCI-H460) (Fig.4A).

To analyze the mitochondrial respiration, a mitochondrial stress test was performed. The OCR was measured during cells’ treatment with: corresponding assay media (Fig. 4B, blue line A); then with olygomycin (Fig. 4B, blue line B); next with FCCP (electron transporter chain accelerator) that is an uncoupling agent which disrupts ATP synthesis leading to the collapse of the mitochondrial membrane potential and causing rapid consumption of energy and oxygen without the generation of ATP (Fig. 4B, blue line C); and finally with rotenone (complex I inhibitor) which shuts down mitochondrial respiration by preventing the transfer of electrons from complex I to coenzyme Q (Fig. 4B, blue line D). These modulators allowed to calculate the following parameters: Non-mitochondrial respiration (= minimum rate measurement after rotenone injection); Basal respiration (= last rate measurement before first injection - non-mitochondrial respiration rate); Maximal respiration (= maximum rate measurement after FCCP injection – non-mitochondrial respiration); Proton leak (= minimum rate measurement after olygomycin injection – non-mitochondrial respiration); ATP production (= last rate measurement before olygomycin injection – minimum rate measurement after olygomycin injection); Spare capacity (= maximal respiration – basal respiration).

Regarding the mitochondrial respiration, the MDR cells showed a statistically significant decrease in the non-mitochondrial respiration, basal respiration, maximal respiration, proton leak, ATP production and spare capacity (Fig. 4B).

### PPP inhibitor changed the metabolic phenotype of NCI-H460 cells towards the phenotype of their MDR counterparts (NCI-H460/R)

**Figure 5:**
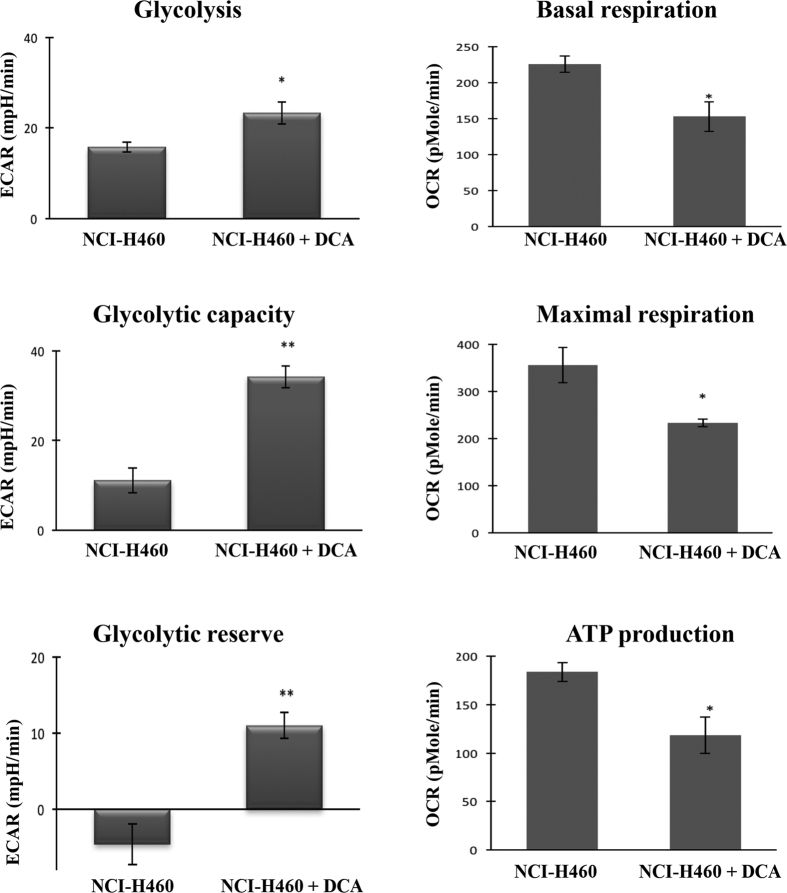
Alterations in NCI-H460 cell metabolism after treatment with a PPP inhibitor. NCI-H460 cells were treated with 5 mM DCA for 24 h and subsequently metabolically profiled, using the *Seahorse XF*-*24 Analyser*. Data represent mean ± SEM from three independent experiments, with four replicates per experiment. *p ≤ *0.05*; **p ≤ *0.01*; NCI-H460 *vs*. NCI-H460+DCA. ECAR - extracellular acidification rate; OCR - oxygen consumption rate.

As aforementioned, sensitive cells possess more rate limiting enzymes of the PPP than their corresponding MDR cells (Tables 1 and 2 and Fig. 2). In addition, they showed a decrease in glycolytic parameters (Fig. 4A) and an increase in mitochondrial respiration (Fig. 4B). In order to confirm that the PPP is involved in the MDR phenotype, NCI-H460 cells (drug-sensitive) were treated with a sub-lethal concentration of dichloroacetate (DCA, a PPP inhibitor) and consequent alterations in glycolysis and OXPHOS were observed. After DCA treatment, the drug-sensitive cells acquired a metabolic phenotype similar to the one observed in the MDR cells, presenting an increase in glycolysis, glycolytic capacity and glycolytic reserve, and a decrease in their basal respiration, maximal respiration and ATP production by OXPHOS (Fig. 5). The MDR cells (NCI-H460/R) treated with DCA showed no changes in glycolytic function and mitochondrial respiration (data not shown).

### P-gp inhibitor changed the metabolic phenotype of MDR cells (NCI-H460/R) towards the phenotype of their corresponding sensitive cells (NCI-H460)

**Figure 6:**
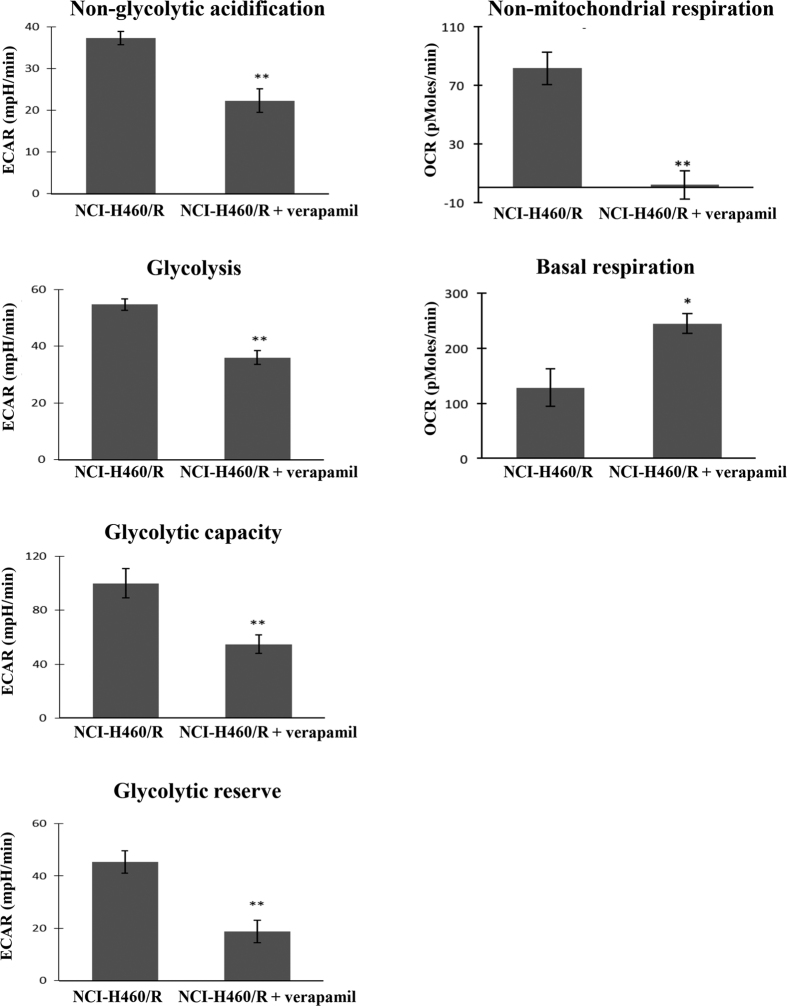
Alterations in the NCI-H460/R cellular metabolism after treatment with a P-gp inhibitor. NCI-H460/R cells were treated with 2 μM verapamil for 15 h and subsequently metabolically profiled using the *Seahorse XF*-*24 Analyser* for measuring alterations in glycolysis, glycolytic capacity, glycolytic reserve, non-glycolytic acidification, basal respiration and non-mitochondrial respiration. Data represent three independent experiments, with four replicates per experiment. *p ≤ *0.05*; **p ≤ *0.01*; NCI-H460/R *vs*. NCI-H460/R+verapamil. ECAR - extracellular acidification rate; OCR - oxygen consumption rate.

In order to confirm the possible influence of P-gp in the observed metabolic alterations of the MDR cells, the NCI-H460/R were treated with a sub-lethal concentration of a well-known P-gp inhibitor, verapamil, and the levels of glycolysis and OXPHOS were analyzed. After treatment with verapamil, the MDR cells acquired a metabolic phenotype more similar to the one observed in sensitive cells, presenting a decrease in glycolysis, glycolytic capacity, glycolytic reserve and non-glycolytic acidification, and an increase in their basal respiration (Fig. 6).

### PPP inhibitor increased resistance of sensitive cells (NCI-H460) to doxorubicin

**Figure 7:**
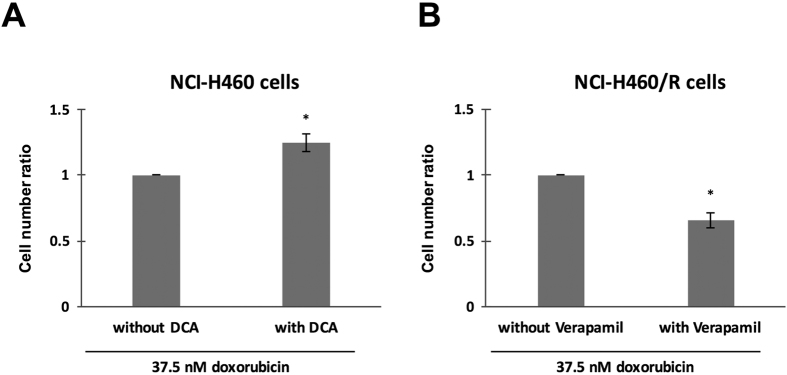
Alterations in the resistance level of NCI-H460 and NCI-H460/R cells after treatment with PPP and P-gp inhibitors, respectively. Cells with or without pre-treatment with DCA or verapamil were treated with 37.5 nM of doxorubicin and the ratio between cells with and without pre-treatment was calculated. (**A**) NCI-H460 cells were pre-treated with 5 mM DCA for 24 h. (**B**)- NCI-H460/R cells were pre-treated with 2 μM verapamil for 15 h. Data represent mean ± SEM from three independent experiments. *p ≤ *0.05*.

To evaluate the possible contribution of PPP to the MDR phenotype, the NCI-H460 cells were pre-treated with a sub-lethal concentration of PPP inhibitor - DCA and then subjected to doxorubicin treatment (37.5 nM). The relative cell number was assessed and the ratio between sensitive cells treated with DCA and without DCA was calculated. The sensitive cells pre-treated with DCA were more resistant to doxorubicin compared to sensitive cells without DCA pre-treatment (Fig. 7A). The increase in doxorubicin resistance was also observed when cells were treated with higher concentrations of doxorubicin (75 nM and 150 nM) (data not shown).

### P-gp inhibitor sensitized MDR cells (NCI-H460/R) to doxorubicin

To confirm the P-gp involvement in the MDR phenotype, the NCI-H460/R cells were pre-treated with a sub-lethal concentration of P-gp inhibitor - verapamil and then subjected to doxorubicin treatment (37.5 nM). The relative cell number was assessed and the ratio between MDR cells treated with verapamil and without verapamil was calculated. The MDR cells pre-treated with verapamil were more sensitive to doxorubicin compared to MDR cells without verapamil pre-treatment (Fig. 7B). The sensitization of MDR cells to doxorubicin was also observed with other concentrations of doxorubicin in a dose dependent manner (75 nM and 150 nM, data not shown).

### EVs from MDR cells were able to transfer their metabolic phenotype to the sensitive cells

**Figure 8:**
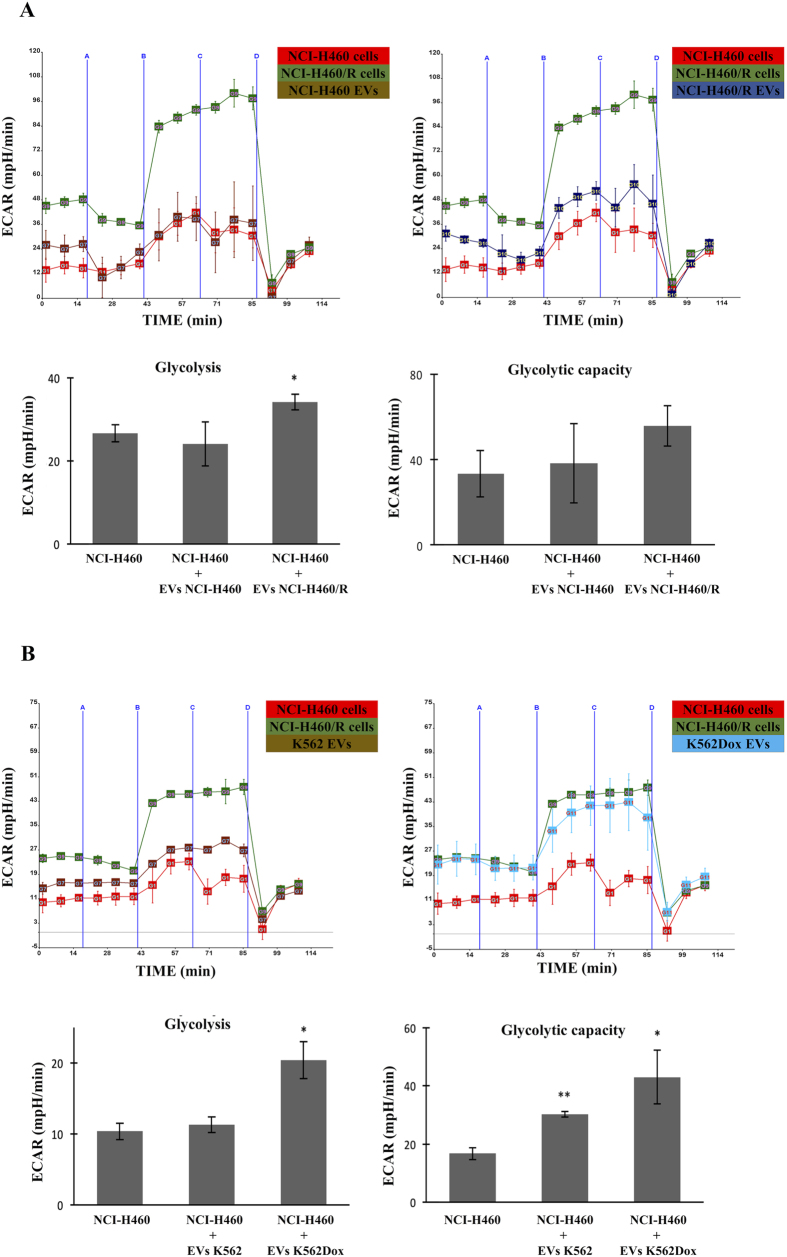
Alterations observed in NCI-H460 cellular metabolism after treatment with EVs shed by MDR cells. NCI-H460 cells were treated with 18 × 10^8^ EVs for 15 h and subsequently metabolically profiled using *Seahorse XF*-*24 Analyser*. The results of glycolysis stress test are shown as ECAR measurements after addition of glucose-free media (blue line A), glucose (blue line B), oligomycin (blue line C) and 2-deoxyglucose (blue line D). (**A**) NCI-H460 cells were treated with their own EVs and with EVs isolated from NCI-H460/R cells. (**B**) NCI-H460 cells were treated with EVs isolated from the pair of chronic myeloid leukaemia counterpart cells, K562 and K562Dox. Data represents the results from two independent experiments, with four replicates each. A two-tailed *p ≤ *0.05*; **p ≤ *0.01*; ***p ≤ *0.001* NCI-H460 *vs*. NCI-H460+EVs NCI-H460; NCI-H460 *vs*. NCI-H460+EVs NCI-H460/R; NCI.H460 *vs*. NCI-H460+EVs K562; NCI-H460 *vs*. NCI-H460+EVs K562Dox.

Next, we wanted to verify if MDR phenotype could be transferred to the drug-sensitive cells via EVs shed by the MDR cells. Therefore, drug-sensitive cells (NCI-H460) were treated with EVs isolated from the counterpart pairs of non-small cell lung cancer cells (NCI-H460 and NCI-H460/R) and chronic myeloid leukemia cells (K562 and K562Dox) (Fig. S1). After 15 h incubation, the drug-sensitive cells treated with the EVs shed by MDR cells (NCI-H460/R - Fig. 8A and K562Dox - Fig. 8B) acquired a metabolic phenotype more similar to the MDR cellular phenotype, *i.e*., an increase in glycolysis and in glycolytic capacity.

## Discussion

Multidrug resistance (MDR) is a major problem in cancer treatment, responsible for chemotherapy failure^4^. MDR cancer cells usually have multiple mechanisms of resistance^14^. One of the most frequent MDR mechanisms is the overexpression of ABC transporters, such as P-gp^2^. Recent findings pointed that altered metabolic pathways help cancer cells to proliferate, adapt their metabolism to nutrient limited conditions, and importantly develop drug resistant phenotypes^22^. Therefore, targeting cellular metabolism could chemosensitize MDR cells^9^,^23^,^24^. Understanding the metabolic adaptations of MDR cancer cells is important for the identification of new approaches to counteract this phenotype.

In the present study, we have identified a complex network of metabolic alterations associated with the MDR phenotype that could lead to the identification of more efficient therapeutic MDR circumvention strategies. In addition, we have shown that EVs released by MDR cells are capable of stimulating a metabolic alteration (towards a MDR phenotype) in recipient drug-sensitive cancer cells.

We confirmed that comparative proteomic approach is a powerful tool to investigate MDR mechanisms in cancer cells. MDR and their corresponding drug-sensitive cell lines from two distinct models (chronic myeloid leukemia and non-small cell lung cancer) were subjected to a label-free LC-MS quantitative proteomics. The obtained data allowed a comparison between the proteome from MDR cells and the one obtained from their drug-sensitive counterparts. In terms of biological processes, most of the identified DEPs (differentially expressed proteins between drug-sensitive and MDR cells) were involved in metabolic processes and the most active pathways enriched were those involved in cellular metabolism (GSH, PPP and Glycolysis). Therefore, we showed that the two MDR cell models acquired a similar metabolic profile but significantly distinct from the metabolic profile observed in their corresponding drug-sensitive cells.

Interestingly, most of the DEPs involved in the PPP (including the rate limiting enzyme, G6PD) were downregulated in the MDR cells. PPP is a pivotal biosynthetic pathway branched to glycolysis and one of the main antioxidant cellular defense systems^25^. PPP has a central role as a source of nucleic acid precursors and provides reducing power and ribose phosphate to the cell^26^. Changes in the PPP activity can affect response to anticancer drugs, however the specific role of PPP in MDR phenotype is still unclear. Other authors implied that PPP is more active in MDR tumors^27^,^28^. Moreover, evidence suggested that elevated levels of NADPH and GSH, together with an active PPP, play an important role in MDR^29^,^30^. Our results together with other recent studies^31^,^32^ contradict the above-mentioned reports, showing that PPP enzymes are downregulated in MDR cells. These results are controversial since one of the roles of PPP is to provide reducing power to the GSH metabolism and high levels of GSH in tumors have been linked to the development of MDR^33^. In addition, we found increased GSH levels in both MDR models, as previously published by other authors^34^,^35^. Moreover, since GSH is a detoxification agent^36^, the levels of ROS in the MDR cells were decreased. In all, our results suggest that MDR cells are capable of maintaining high GSH levels even when PPP is downregulated. Therefore, we assumed that MDR cells upregulated another source of GSH.

The methionine cycle is a key pathway for many methylation reactions (methylation of DNA, histones and non-histone proteins, such as transcription factors) and could be the source of cysteine residues necessary for GSH synthesis^37^. Growing evidence links aberrant regulation of methylation to tumorigenesis^38^. The epigenetic mechanisms underlying drug resistance have not been fully elucidated, although some studies have suggested the contribution of an altered chromatin state to drug resistance^39^. Our results showed considerable differences between MDR and their drug-sensitive cells, in the amount of several metabolites of the methionine/SAMe pathway. Methionine levels were significantly increased in the MDR cells, which could lead to the GSH increase observed in those cells. In addition, the methylation capacity of the cells was increased in both MDR models and this could be attributed to the metabolic alterations in MDR cells during development of their resistant phenotype. In agreement with our results, other authors have described that treatment with a methylation inhibitor reversed drug resistance indicating that the development of some cases of drug resistance could be methylation-dependent^40^. Also, MTA, dcSAMe, betaine, choline and serine are altered in MDR cells, although not in a similar manner. These differentially altered metabolites indicate that among these two MDR models there are also metabolic differences, which enhance the idea of the complex metabolism associated to MDR.

The energy dependence of drug transport in MDR cells suggests that during development of MDR, cells undergo alterations in the energy utilization pathways. The main cell energy carrier, ATP, is produced in two metabolic pathways: glycolysis and/or oxidative phosphorylation (OXPHOS) of metabolic fuels^22^. One of the fundamental hallmarks of cancer cells is the shift in the balance between these two energy-production pathways, in favor to glycolysis, known as the Warburg effect^41^,^42^,^43^. For this reason, glycolysis inhibition has attracted significant interest as a possible way to sensitize cancer cells to chemotherapy. Indeed, several studies in different cancer models have demonstrated an efficient suppression of MDR by glycolytic inhibitors^8^,^23^,^24^,^44^,^45^. Moreover, during the course of this work another study has suggested the possibility of an accelerated process of glycolysis in MDR cells to increase their energy supply^46^. To our knowledge, a comparison between MDR and drug-sensitive cells in terms of metabolic energy supply, using different modulators of glycolysis and OXPHOS, has never been performed. The results presented herein showed a statistically significant increase in glycolysis and in the glycolytic capacity of the MDR cells (NCI-H460/R), together with a decrease in the mitochondrial basal respiration and in the maximal capacity of the cells to perform OXPHOS compared to the drug-sensitive counterparts (NCI-H460). Therefore, our findings indicate that MDR cells, when acquiring the resistant phenotype, enhance their switch in the cellular energy supply pathways from OXPHOS to glycolysis. This altered metabolic activity in MDR cells could be crucial for: (i) supporting uncontrolled proliferation, since glycolysis provides the intermediates necessary for biosynthetic pathways; (ii) allowing the use of the most abundant extracellular nutrient (glucose) to produce abundant ATP. Even though the yield of ATP per glucose consumed is low, if the glycolytic flux is high enough, the percentage of cellular ATP produced by glycolysis can exceed the one produced by OXPHOS^47^,^48^. Therefore, this metabolic phenotype could be beneficial for MDR cells at both levels of bioenergetics and biosynthesis.

Although MDR cells perform less OXPHOS, the application of different OXPHOS modulators (oligomycin, FCCP and rotenone) affected more the drug-sensitive than their MDR counterpart cells. The OCR levels in the MDR cells have been less affected than the OCR levels in drug-sensitive cells. This suggests that MDR cells are more capable of sustaining the acquired metabolic phenotype in order to maintain the higher energy demand. In accordance, it was reported that the relationship between drug resistance and glycolysis may partially be due to the radical scavenging potential of the glycolytic intermediates, and the link between them and the cellular redox status^49^.

Furthermore, the increase in glycolysis could be responsible for the decrease in the PPP observed in the MDR cells. Since some of the glycolysis and PPP intermediates are the same, it is possible that MDR cells are directing these intermediates mostly to glycolysis, in order to sustain the energy for the requested biosynthetic pathways. In fact, we demonstrated that when the PPP pathway was inhibited (with DCA^50^), drug-sensitive cells (NCI-H460) acquired a metabolic phenotype similar to that observed in MDR cells by increasing glycolysis and decreasing OXPHOS. Also, PPP inhibition increased resistance of sensitive cells to doxorubicin, when compared to sensitive cells without PPP inhibition. These results support the hypothesis that there is a competition between glycolysis and PPP activity in the MDR phenotype and also that modulation of the PPP and glycolytic pathways is capable of altering the sensitivity of cells to drugs.

Additionally, in multicellular tumor spheroids and in doxorubicin-resistant human breast adenocarcinoma cells, the inhibition of glycolysis raised intracellular ROS, downregulated P-gp and reverted the MDR phenotype^8^,^23^. In agreement with these data, our work showed that treatment of MDR cells (NCI-H460/R) with a P-gp inhibitor (verapamil) switched their metabolic phenotype to that characteristic of sensitive cells (decrease in glycolysis and an increase in OXPHOS) and also promoted sensitization of the MDR cells to doxorubicin.

Several studies have associated metabolic alterations with the shedding and cargo of EVs in different types of cells (such as increase lactate production^18^, inhibition of glutamine metabolism^19^ and the presence of several enzymes involved in glucose and glutamine metabolism^20^). The importance of EVs in the transfer of the MDR phenotype has been recently described^13^,^14^,^51^. Therefore, the acquisition by MDR cells of a different metabolic phenotype, could be responsible for the differences in: (i) the type of EVs population released by MDR cells; (ii) their cargo and (iii) the capacity to transfer phenotypes to receiving cells. In fact, we have recently shown that MDR cells shed a different population of EVs (more microvesicle-like EVs and less exosomes) when compared to the EVs shed by the drug-sensitive counterpart cells^17^,^52^. Additionally, our results showed that drug-sensitive cells (NCI-H460) co-incubated with EVs from MDR cells (NCI-H460/R and K562Dox), acquired a metabolic phenotype (increase in glycolysis) similar to the one observed in MDR cells. These results showed that, independently of the origin of the MDR cells (leukemia or lung cancer), their EVs are capable of inducing such alterations. However, surprisingly, our results showed that the EVs from the sensitive leukemic cell line were also capable of inducing alterations in the glycolytic capacity of the recipient drug-sensitive cells. The reason for this is unknown, but is possibly due to the exposure of cells to high levels of EVs, therefore not related to MDR. Furthermore, a recent study has described that glutamine metabolism was altered in cancer cells following incubation with large EVs (microvesicles), an effect that was not observed upon incubation with exosomes^20^. This further highlights the importance of verifying the type of EVs released by donor cells, since different EVs may transfer different phenotypes to receiving cells.

## Conclusions

**Figure 9:**
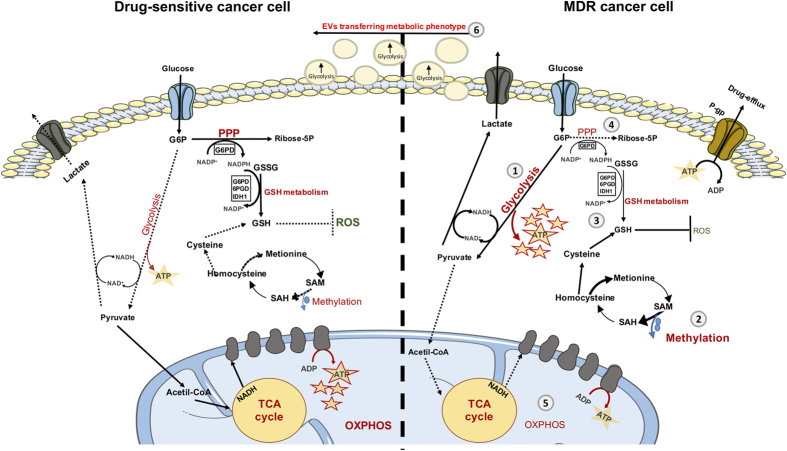
Schematic representation of the observed metabolic differences between drug-sensitive cells and their counterpart MDR cells. P-gp overexpressing MDR cancer cells have various protective metabolic strategies. These include: increasing rates of glycolysis (1) and methylation capacity (2), alterations in the GSH metabolism (3), decreasing rates of PPP (4) and OXPHOS (5) and finally changing the phenotype of the surrounding drug-sensitive cells by EVs-mediated transfer of new features (6). Filled lines and arrows: increased pathways; Dashed lines and arrows: decreased pathways; Bold and bigger fonts: increased metabolic processes; Smaller fonts: decreased metabolic processes.

In conclusion, P-gp overexpressing MDR cells may employ various protective metabolic strategies to survive (schematically represented in Fig. 9). These include (i) alterations in the GSH metabolism, (ii) increasing the methylation index influencing epigenetic regulation, (iii) increasing the rates of glycolysis and (iv) changing the phenotype of the surrounding drug-sensitive cells, by EVs mediated transfer of new features. Our results indicate that the development of MDR is a complex phenomenon that involves several simultaneous metabolic alterations.

We clarified for the first time the complex metabolic network of the various metabolic alterations associated with MDR in cancer cells. In addition, that MDR cancer cells are more capable than drug-sensitive cells of sustaining its specific metabolic profile. Specifically, we showed for the first time that MDR cancer cells, besides increased glycolysis also have increased glycolytic capacity and glycolytic reserve, while their OXPHOS rate is decreased. In addition, our work found differences between MDR and drug-sensitive cells in the amount of several metabolites of the methionine/SAMe pathway which regulates DNA and protein methylation, as well as GSH production. Finally, we demonstrated for the first time the transfer of metabolic information from MDR to drug-sensitive cancer cells through a specific population of EVs.

## Materials and Methods

### Cell culture

The chronic myeloid leukemia cell line K562 was from *European Collection of Cell Cultures* (ECACC) and its P-gp overexpressing counterpart cell line K562Dox was a kind gift of Dr. J.P. Marie (Paris, France)^53^,^54^. The non-small cell lung cancer cell line NCI-H460 and its drug-resistant P-gp overexpressing counterpart cell line NCI-H460/R were a kind gift from Dr. M. Pešić (Belgrade, Serbia)^55^,^56^. All cell lines were genotyped and routinely monitored for mycoplasma contamination by PCR (VenorGeM^®^ Advance Mycoplasma Detection Kit, Minerva). All cells were routinely grown in RPMI-1640 (with Ultraglutamine I and 25 mM HEPES) medium (Lonza), supplemented with 10% fetal bovine serum (FBS, PAA) at 37 °C in a humidified incubator with 5% CO_2_ in air. Cell number and viability were analyzed with trypan blue exclusion assay. All experiments were carried out with exponentially growing cells having over 90% viability.

### Sample preparation and mass spectrometry using LC/MS/MS

Pellets from cells (K562, K562Dox, NCI-H460 and NCI-H460/R) were processed and 10 μg of proteins were analyzed by mass spectrometry using LC/MS/MS as previously described^17^. Biological replicates (from 4 independent experiments) were analyzed for each sample type.

### Label-Free LC-MS quantitative profiling

Label-free LC–MS analysis was carried out using *Progenesis QI* for proteomics v4.1 software (NonLinear Dynamics, UK), essentially as recommended by the manufacturer (see www.nonlinear.com for further background to alignment, normalization, calculation of peptide abundance, etc.).

This software extracts quantitative information from MS1 data by aligning the data based on the LC retention time of each sample to a reference file (sample run that yielded most peptide ions); this allows for any drift in retention time, giving an adjusted retention time for all runs in the analysis.

Results were filtered, based on statistical analysis. The *Progenesis* peptide quantification algorithm calculates peptide abundance as the sum of the peak areas within its isotope boundaries. Each abundance value is then transformed to a normalized abundance value by applying a global scaling factor. Protein abundance was calculated as the sum of the abundances of all peptide ions, which have been identified as coming from the same protein. Any peptides with an ANOVA score of p > 0.05 were eliminated. The MS2 data for the remaining peptides was exported and the resulting MGF file used to search the NewUniProtSwissprot database (updated in January 2014) on the MASCOT server (www.matrixscience.com) for protein identifications. The search parameters used were as follows: (1) species, Homo sapiens; (2) allowed number of missed cleavages, 2; (3) fixed modification, carboxylmethyl; (4) variable modifications, methionine oxidation; (5) peptide mass tolerance ± 20 ppm; (6) MS/MS tolerance ± 0.6 Da; and (7) peptide charge +1, +2 and +3. Only peptides with ion scores of 40 and above were considered and re-imported back into *Progenesis QI* software for further analysis. Peptide identifications were imported into the *Progenesis* software and assigned to the matching features. Protein scores were based on ANOVA values with a cut off of p < 0.05. Differentially expressed proteins (DEPs) between drug-sensitive and MDR cells with ≥2 peptides matched and a ≥ 1.5 fold differences in abundance were considered as significant. Peptide conflicts occur when a peptide is identified as present in more than one protein. These were resolved by assigning the peptide to the protein with the greater number of hits, a greater Mascot score or a lower mass error; when conflicts could not be clearly resolved, the peptide was excluded from the analysis.

### Bioinformatics analysis of the detected DEPs

The two data sets (DEPs from K562 *vs*. K562Dox cells and NCI-H460 *vs*. NCI-H460/R cells) were analyzed using bioinformatics methods. Database for Annotation, Visualization, and Integrated Discovery (DAVID 6.7) was used to identify protein and molecular pathway modifications. UniProt accession numbers were obtained by *Progenesis* software. To understand the high-level functions and utilization of the biological systems from molecular-level information, the *Kyoto Encyclopedia of Genes and Genomes (KEGG*) pathway was used. Distributions in subcellular locations, biological processes and molecular functions were assigned to each protein based on *Gene Ontology (GO*) categories. The significantly (p < 0.05) enriched categories are presented.

### Analysis of Protein Expression by Western Blot

Cell pellets were lysed in Winman’s Buffer (1% NP-40, 0.1 M Tris-HCl pH 8.0, 0.15 M NaCl and 5 mM EDTA) with EDTA-free protease inhibitor cocktail (Roche). Total protein content of cell lysates was quantified with “DC Protein assay kit” (Bio-Rad) and 20 μg of protein were subjected to SDS-PAGE (12% Bis-Tris gel). Following electrophoretic transfer of the proteins into nitrocellulose membranes (GE Healthcare, UK), membranes were then incubated with the following primary antibodies: goat anti-Actin (1:2000; Santa Cruz Biotechnology), mouse anti-P-gp (P7965) (1:2000; Sigma), G6PD (1:200; Santa Cruz Biotechnology), 6PGD (1:200; Santa Cruz Biotechnology) and IDH1 (1:200; Santa Cruz Biotechnology). The following secondary antibodies were then used: anti-mouse IgG-HRP; anti-rabbit IgG-HRP or anti-goat IgG-HRP (all diluted 1:2000; Santa Cruz Biotechnology). Signal was detected using the ECL Western blot Detection Reagents (GE Healthcare, UK), the Amersham Hyperfilm ECL (GE Healthcare, UK), and the Kodak GBX developer and fixer (Sigma, EUA)^57^. The intensity of the bands obtained in each film was further analyzed using the software Quantity One – 1D Analysis (Bio-Rad, USA).

### GSH/GSSG-Glo^TM^ assay

To measure GSH levels, the GSH/GSSG-Glo^TM^ Assay (Promega, USA) was used following manufacturer’s instructions. Briefly, 5000 cells/well were plated in white 96-opaque well plates. NCI-H460 and NCI-H460/R cells were plated in 100 μl of RPMI-1640; K562 and K562Dox cells were plated in 20 μl of Hanks Balanced Salt Solution (HBSS). Total glutathione lysis reagent or oxidized glutathione lysis reagent were added to the cells. After incubation at room temperature a luciferin generation reagent was added to all samples and the plates were incubated for 30 min at room temperature. Luminescence was quantified using a microplate reader (Biotek Instruments Inc. Synergy MX. USA). Positive control (cells treated with 5 μM staurosporine for 4 h) and wells with no cells were also included as controls. GSH levels were calculated as follows: GSH levels = total glutathione levels – oxidized glutathione levels.

### ROS Detection

2′,7′-dichlorodihydrofluorescein diacetate (H*2*DCF-DA) analysis by flow cytometry were used to measure ROS concentration in drug-sensitive and MDR cells. K562 and K562Dox cells (8 × 10^5^/well) were plated in 6-well plates; NCI-H460 and NCI-H460/R cells (3 × 10^5^/well) were plated in 6-well plates for 24 h. Cells were harvested and incubated in adequate medium with 10 μM CM-H2DCFDA for 45 min at 37 °C in the dark. Cells were subsequently washed twice in PBS and the CM-H2DCFDA fluorescence was analyzed in a BD Accuri flow cytometer (FL1-H channel). A positive control (cells treated with 1 μM staurosporine for 6 h) was also included. Mean fluorescence intensity was calculated after correction for auto-fluorescence.

### Semi-quantitative analysis of metabolites of the methionine/S-adenosylmethionine pathway

Total cell pellets (4 independent preparations for each cell line) were lysed in 500 μL of a mixture of ice-cold water/metanol/10 mM acetic acid (49/50/1 v/v/v%) with a tissue homogenizer (Precellys) in 1 × 20 second cycles at 6000 rpm. Subsequently, 400 μL of the homogenate was transferred to a new aliquot and shaken at 1400 rpm for 30 minutes at 4 °C. Next the aliquots were centrifuged for 15 min at 14000 rpm at 4 °C. 75 μL of the supernatant was transferred to a fresh aliquot and placed at −80 °C for 20′. The chilled supernatants were evaporated with a speedvac in approximately 3 h. The resulting pellets were resuspended in 100 μL water/acetonitrile (MeCN)/formic acid (40/60/0.1 v/v/v%, resuspension solution).

Concentrations of methionine, MTA, SAMe, SAH, spermidine and spermine were determined with a semi-quantitative method. Calibration curves for these compounds were obtained, by measuring serial dilutions of a pooled standard mixture in resuspension solution. The concentrations for all compounds in the dilutions ranged from 100 μM to 0.025 μM. For the standard mixtures, separate 10 mM stocks of the standards were made. These were then pooled and further diluted in resuspension solution in order to obtain the final concentrations as used for the calibration curve.

Samples were measured with a UPLC system (Acquity, Waters, Manchester) coupled to a Time of Flight mass spectrometer (ToF MS, SYNAPT G2, Waters). A 2.1 × 100 mm, 1.7 μm BEH amide column (Waters), thermostated at 40 °C, was used to separate the analytes before entering the MS. Solvent A (aqueous phase) consisted of 99.5% water, 0.5% formic acid and 20 mM ammonium formate while solvent B (organic phase) consisted of 29.5% water, 70% MeCN, 0.5% formic acid and 1 mM ammonium formate.

In order to obtain a good separation of the analytes the following gradient was used: from 5% A to 50% A in 2.4 minutes in curved gradient (#8, as defined by Waters), from 50% A to 99.9% A in 0.2 minutes constant at 99.9% A for 1.2 minutes, back to 5% A in 0.2 minutes. The flow rate was 0.250 mL/min and the injection volume was 2 μL. All samples were injected randomly. After every 10 injections a QC sample was injected. All samples were injected in duplicate.

Analytes were measured in enhanced duty cycle (EDC) mode, optimized for the mass of the analyte in question. MTA was measured in scan function 1 (EDC at 298), choline was measured in scan function 2 (EDC at 104), methionine was measured in scan function 3 (EDC at 150), SAH was measured in scan function 8 (EDC at 385), SAMe was measured in scan function 10 (EDC at 399), spermidine was measured in scan function 12 (EDC at 146), spermine was measured in scan function 13 (EDC at 203). The cone voltage was between 20 and 25 depending on the analyte. A 2 ng/mL leucine-enkephalin solution in water/acetonitrile/formic acid (49.9/50/0.1%v/v/v) was infused at 10 μL/min and used for a lock mass which was measured each 47 seconds for 0.5 seconds. Spectral peaks were automatically corrected for deviations in the lock mass.

Extracted ion traces were obtained for methionine (*m/z* = 150.0589), SAH (*m/z* = 385.1294), SAMe (*m/z* = 399.1451), MTA (*m/z* = 298.097), spermidine (*m/z* = 146.1657) and Spermine (*m/z* = 203.2236) in a 20 mDa window and subsequently smoothed (2 points, 2 iterations) and integrated with QuanLynx software (Waters, Manchester).

### Extracellular flux assay using Seahorse XF-24 Analyser

ECAR, reflecting the rate of glycolysis, and OCR, reflecting the rate of OXPHOS, were measured using a *Seahorse Bioscience* (Copenhagen, Denmark) XF24 analyzer. Briefly, 2 × 10^4^ cells/well for glycolysis stress test and 3 × 10^4^ cells/well for mitochondrial stress test were plated in *Seahorse XF24* plates in 200 μl of RPMI-1640 and incubated for 20–24 h at 37 °C in a humidified incubator with 5% CO_2_ in air and 1 h hour prior to the XF assay in a humidified incubator without CO_2_ with the corresponding assay media. In the experiments where the cells were treated with DCA, the treatment was performed for 24 h with a concentration of 5 mM. The verapamil treatment was performed for 15 h with 2 μM. NCI-H460/R treated with DCA and NCI-H460 treated with verapamil were used as controls.

Basal OCR measurements were made in DMEM containing 10 mM sodium pyruvate (Invitrogen, California, EUA), 10 mM Glucose (Invitrogen, California, EUA) and 10 mM Glutamax (Invitrogen, California, EUA) and Basal ECAR measurements were made in DMEM without any supplementation.

Steady-state (baseline) oxygen consumption rates and extracellular acidification rates were measured. Non-glycolytic acidification, glycolysis, glycolytic capacity and glycolytic reserve were measured, through ECAR, by injecting: glucose, oligomycin and 2-deoxyglucose. Basal respiration, proton leak, spare capacity, maximal respiration, non-mitochondrial respiration and ATP production were measured, through OCR, after the sequential injection of oligomycin, FCCP and rotenone. All measurements were normalized to protein quantity with crystal violet.

### Sulforhodamine B assay

The Sulforhodamine B (SRB) assay was performed to measure cell growth indirectly, by measuring total protein content. The NCI-H460 and NCI-H460/R cells were seeded in 96-well plates (5000 cells/well), and pre-treated with 5 mM DCA for 24 h or with 2 μM verapamil for 15 h, respectively. Cells were then treated in duplicate with 3 serial dilutions of doxorubicin (37.5 nM, 75 nM and 150 nM) for 48 h. Cells were fixed with 10% (w/v) ice cold trichloroacetic acid (TCA) and then washed with distilled water. After staining proteins with 0.4% (w/v) SRB, cells were washed with 1% (v/v) acetic acid, the bound SRB was solubilized with 10 mM Tris base and absorbance was measured at 510 nm in a multiplate reader (Synergy Mx, Biotek Instruments Inc.). The ratio between cells with a pre-treatment and cells without the pre-treatment was calculated. NCI-H460 treated with DCA only and NCI-H460/R treated with verapamil only were used as controls.

### Co-culture of H460 cells with EVs

EVs were isolated from the culture media of drug-sensitive or MDR cells by various centrifugation steps as previously described^17^. EVs pellets were re-suspended in 100 μl of PBS and frozen at −80 °C. NCI-H460 cells were treated with 18 × 10^8^ EVs (quantified by nanoparticle tracking analysis as in Supplementary Information) for 15 hours and were subsequently metabolically profiled using *Seahorse XF*-*24 Analyzer*.

### Statistical analysis

All presented data resulted from at least three independent experiments (excluding the EVs treatment which was performed in two independent experiments only). All data was statistically analyzed with the two-tailed unpaired Student’s *t*-test. Results were considered statistically significant when *p* ≤ 0.05.

## Additional Information

**How to cite this article:** Lopes-Rodrigues, V. *et al*. Identification of the metabolic alterations associated with the multidrug resistant phenotype in cancer and their intercellular transfer mediated by extracellular vesicles. *Sci. Rep.* **7**, 44541; doi: 10.1038/srep44541 (2017).

**Publisher's note:** Springer Nature remains neutral with regard to jurisdictional claims in published maps and institutional affiliations.

## Supplementary Material

Supplementary Information

## Figures and Tables

**Table 1:** Metabolic pathways found enriched with DEPs in MDR cells.

	Term	Count^a^	ANOVA (P)	Genes Symbol
K562 *vs*.K562Dox	Glutathione metabolism	4	0.0086	G6PD, PRDX2, IDH1, 6PGD
	Pentose phosphate pathway	3	0.0025	G6PD, ALDOC, 6PGD
	Glycolysis	2	0.01	ALDOC, PKM2
NCI-H460 *vs*.NCI-H460/R	Glutathione metabolism	5	0.00064	G6PD, 6PGD. IDH1, MGST1, PRDX2
	Pentose phosphate pathway	4	0.00099	G6PD, 6PGD, TKT, TALDO1
	Glycolysis	1	nd	ALDH3A1

Results were analyzed with DAVID software for KEGG pathway enrichment.

^a^Number of proteins involved in each pathway.

nd – no data.

**Table 2:** DEPs involved in MDR and metabolic processes.

		UniProt accession no.	Protein description	Gene Symbol	Peptides	Mascot Score	ANOVA (P)	Fold Change^a^
K562 *vs*. K562Dox	*Increased in MDR cells*	P08183	P-glycoprotein	MDR1	6	404.08	1.24e-009	18.80
		Q9Y4L1	Hypoxia up-regulated protein 1	HYOU1	4	282.01	3.19e-005	2.05
		Q92597	Protein NDRG1	NDRG1	2	118.12	4.32e-006	1.84
		P53396	ATP-citrate synthase	ACLY	5	289.70	7.19e-006	1.84
		P14618	Pyruvate kinase	PKM2	10	706,77	4,25E-03	1.52
		P11586	C-1-tetrahydrofolate synthase	MTHFD1	2	97,89	9,83E-04	1.51
	*Decreased in MDR cells*	P09972	Fructose-bisphosphate aldolase C	ALDOC	7	550.98	0.01	0.66
		P52209	6-phosphogluconate dehydrogenase	6PGD	2	111,38	2,84E-03	0.65
		O75874	Isocitrate dehydrogenase	IDH1	3	218,56	2,63E-05	0.65
		P32119	Peroxiredoxin-2	PRDX2	5	372.45	1.35e-003	0.60
		P11413	Glucose-6-phosphate 1-dehydrogenase	G6PD	4	250.70	7.79e-007	0.39
NCI-H460 vs. NCI-H460/R	*Increased in MDR cells*	P08183	Multidrug resistance protein 1	MDR1	2	175.04	9.14E-06	64.52
		P10620	Microsomal glutathione S-transferase 1	MGST1	2	105,19	1.33e-004	1.80
		P37837	Transaldolase	TALDO1	2	116.98	4.64e-004	1.53
	*Decreased in MDR cells*	O75874	Isocitrate dehydrogenase	IDH1	4	327.28	7.79E-05	0.66
		Q99798	Aconitate hydratase, mitochondrial	ACO2	2	132,35	2.38e-003	0.63
		P29401	Transketolase	TKT	8	489.12	7.25e-005	0.60
		P52209	6-phosphogluconate dehydrogenase	6PGD	7	570.89	3.89E-06	0.54
		P32119	Peroxiredoxin-2	PRDX2	2	133.35	0.02	0.52
		P11413	Glucose-6-phosphate 1-dehydrogenase	G6PD	13	1012.11	1.22E-06	0.41
		P30838	Aldehyde dehydrogenase	ALDH3A1	6	352,67	1.74e-005	0.40

^a^MDR cells/Drug-sensitive cells.

**Table 3:** Semi-quantitative analysis of metabolites of the methionine/SAMe pathway in two pairs of MDR and their drug-sensitive counterpart cell lines.

	Metabolite description	Fold change^a^	ANOVA (P)
K562 *vs*. K562Dox	Methionine	1.54	2,25E-05
	5′-deoxy-5′-methylthioadenosine (MTA)	1.44	8,37E-04
	S-adenosylhomocysteine (SAH)	0.57	7,67E-06
	S-adenosylmethionine (SAMe)	0.99	8,49E-01
	Spermidine	0.85	3,68E-03
	Betaine	1.02	4,79E-01
	Choline	0.63	3,48E-06
	Decarboxylated S-adenosyl methionine (dc-SAMe)	1.42	4,72E-05
	Serine	0.50	0
	Threonine	0.99	8,59E-01
NCI-H460 vs. NCI-H460/R	Methionine	1.91	3,55E-05
	5′-deoxy-5′-methylthioadenosine (MTA)	1.13	1,44E-01
	S-adenosylhomocysteine (SAH)	0.25	2,42E-05
	S-adenosylmethionine (SAMe)	0.99	7,85E-01
	Spermidine	0.81	1,33E-02
	Betaine	1.71	2,78E-04
	Choline	5.94	2,54E-07
	Decarboxylated S-adenosyl methionine (dc-SAMe)	0.50	1,79E-04
	Serine	1.04	1,78E-01
	Threonine	0.99	8,15E-01

^a^MDR cells/drug-sensitive cells.
