# Supplementary material for: Identification of the metabolic alterations associated with the multidrug resistant phenotype in cancer and their intercellular transfer mediated by extracellular vesicles
Source: Sci Rep. 2017 Mar 17;7:44541. doi: 10.1038/srep44541 (PMC5356019; doi:10.1038/srep44541)
Supplement: Supplementary Information [file srep44541-s1.pdf]

# Supplementary Information

# Identification of the metabolic alterations associated with the multidrug resistant phenotype in cancer and their intercellular transfer mediated by extracellular vesicles

Vanessa Lopes-Rodrigues[1,2,3], Alessio Di Luca[4], Justyna Mleczko[5], Paula Meleady[4], Michael Henry[4], Milica Pesic[6], Diana Cabrera[5], Sebastiaan van Liempd[5], Raquel T. Lima[1,2,7], Robert O'Connor[4], Juan M. Falcon-Perez[5,8], M. Helena Vasconcelos[1,2,9*]

# Material and Methods

## *Nanoparticle Tracking Analysis*

A NanoSight LM10 system (Malvern, U.K.) equipped with a fast video capture and particle-tracking software was used to measure the rate of Brownian motion. All settings for the camera were fixed and kept constant for all measurements during the session. For each sample, at least five videos of 30–60 s with more than 200 detected tracks per video, and in at least one dilution, were taken and analyzed using the Nanoparticle Tracking Analysis software to obtain the mean, mode, median vesicle size and an estimation of the particle concentration. Results represent the mean of all videos acquired for a given sample.

**Table S1 | Statistically significant** DEPs identified with the progenesis software when comparing K562 cells with the K562Dox cells.

| Accession | Peptides | Score | ANOVA (P) | Highest mean condition[a] | Description |
|---|---|---|---|---|---|
| P02100 | 12 | 1151.5 | 7,85E-10 | K562 | Hemoglobin subunit epsilon |
| P08183 | 6 | 404.08 | 1,24E-09 | K562Dox | Multidrug resistance protein 1 |
| Q96AE4 | 3 | 169.33 | 6,60E-09 | K562 | Far upstream element-binding protein 1 |
| P02008 | 6 | 671.58 | 5,46E-08 | K562 | Hemoglobin subunit zeta |
| Q5JQC4 | 2 | 116.32 | 7,57E-08 | K562 | Cancer/testis antigen 47A |
| P13667 | 9 | 637.04 | 8,40E-08 | K562Dox | Protein disulfide-isomerase |
| P21333 | 17 | 1078.45 | 9,94E-08 | K562 | Filamin-A |
| Q96GU1 | 3 | 254.71 | 1,43E-07 | K562 | P antigen family member 5 |
| P07237 | 8 | 477.65 | 1,48E-07 | K562Dox | Protein disulfide-isomerase |
| Q14315 | 7 | 448.29 | 1,73E-07 | K562 | Filamin-C |
| P41252 | 2 | 103.29 | 1,77E-07 | K562Dox | Isoleucine--tRNA ligase, cytoplasmic |
| Q8NBS9 | 4 | 220.76 | 2,75E-07 | K562Dox | Thioredoxin domain-containing protein 5 |
| P69905 | 3 | 333.21 | 3,53E-07 | K562 | Hemoglobin subunit alpha |
| P08107 | 10 | 858.34 | 5,35E-07 | K562 | Heat shock 70 kDa protein 1A/1B |
| P13489 | 5 | 395.69 | 6,32E-07 | K562 | Ribonuclease inhibitor |
| Q8WUM4 | 6 | 340.95 | 6,84E-07 | K562 | Programmed cell death 6-interacting protein |
| P11413 | 4 | 250.7 | 7,79E-07 | K562 | Glucose-6-phosphate 1-dehydrogenase |
| P50402 | 2 | 112.04 | 9,19E-07 | K562 | Emerin |
| Q53FZ2 | 2 | 130.62 | 1,28E-06 | K562Dox | Acyl-coenzyme A synthetase ACSM3, mitochondrial |
| Q15758 | 2 | 103.85 | 1,50E-06 | K562 | Neutral amino acid transporter B(0) |
| P14625 | 11 | 707.02 | 1,56E-06 | K562Dox | Endoplasmin |
| P14314 | 6 | 431.33 | 1,65E-06 | K562Dox | Glucosidase 2 subunit beta |

| | | | | | |
|---|---|---|---|---|---|
| P51659 | 2 | 133.11 | 1,77E-06 | K562Dox | Peroxisomal multifunctional enzyme type 2 |
| P11310 | 2 | 133.95 | 1,97E-06 | K562 | Medium-chain specific acyl-CoA dehydrogenase, mitochondrial |
| Q15084 | 6 | 478.14 | 2,15E-06 | K562Dox | Protein disulfide-isomerase A6 |
| Q9BXL5 | 3 | 203.27 | 2,59E-06 | K562 | Hemogen |
| P04350 | 4 | 262.01 | 2,87E-06 | K562Dox | Tubulin beta-4A chain |
| O75369 | 4 | 259.91 | 2,95E-06 | K562 | Filamin-B |
| Q9UJZ1 | 4 | 319.38 | 3,22E-06 | K562Dox | Stomatin-like protein 2, mitochondrial |
| Q9Y6G9 | 2 | 134.98 | 3,33E-06 | K562Dox | Cytoplasmic dynein 1 light intermediate chain 1 |
| P69892 | 11 | 1152.26 | 3,45E-06 | K562 | Hemoglobin subunit gamma-2 |
| Q9Y2B0 | 2 | 117.53 | 3,65E-06 | K562Dox | Protein canopy homolog 2 |
| P30101 | 13 | 860.94 | 3,88E-06 | K562Dox | Protein disulfide-isomerase A3 |
| P50454 | 7 | 483.96 | 3,90E-06 | K562Dox | Serpin H1 |
| P11021;O95399 | 21 | 1732.04 | 3,96E-06 | K562Dox | 78 kDa glucose-regulated protein |
| Q92597 | 2 | 118.12 | 4,32E-06 | K562Dox | Protein NDRG1 |
| P21980 | 3 | 167.42 | 4,51E-06 | K562 | Protein-glutamine gamma-glutamyltransferase 2 |
| Q02809 | 2 | 112.2 | 4,86E-06 | K562Dox | Procollagen-lysine,2-oxoglutarate 5-dioxygenase 1 |
| P40121 | 4 | 231.66 | 5,10E-06 | K562Dox | Macrophage-capping protein |
| P04083 | 4 | 342.1 | 5,57E-06 | K562Dox | Annexin A1 |
| P30040 | 2 | 96.26 | 7,00E-06 | K562Dox | Endoplasmic reticulum resident protein 29 |
| P53396 | 5 | 289.7 | 7,19E-06 | K562Dox | ATP-citrate synthase |
| O00584 | 2 | 209.89 | 7,98E-06 | K562Dox | Ribonuclease T2 |
| Q9BVA1 | 3 | 201.5 | 9,89E-06 | K562Dox | Tubulin beta-2B chain |
| P02794 | 5 | 371.7 | 9,99E-06 | K562 | Ferritin heavy chain |
| Q15008 | 2 | 105.05 | 1,12E-05 | K562 | 26S proteasome non-ATPase regulatory subunit 6 |
| P23284 | 11 | 645.7 | 1,36E-05 | K562Dox | Peptidyl-prolyl cis-trans isomerase B |

| | | | | | |
|---|---|---|---|---|---|
| P69891 | 12 | 1201.47 | 1,60E-05 | K562 | Hemoglobin subunit gamma-1 |
| P32322 | 3 | 167.48 | 1,98E-05 | K562Dox | Pyrroline-5-carboxylate reductase 1, mitochondrial |
| Q15942 | 2 | 128.16 | 3,04E-05 | K562 | Zyxin |
| P49915 | 3 | 168.86 | 3,09E-05 | K562 | GMP synthase [glutamine-hydrolyzing] |
| Q03252 | 2 | 139.3 | 3,19E-05 | K562Dox | Lamin-B2 |
| Q9Y4L1 | 4 | 282.01 | 3,19E-05 | K562Dox | Hypoxia up-regulated protein 1 |
| P27824 | 5 | 349.95 | 3,35E-05 | K562Dox | Calnexin |
| P13639 | 10 | 667.85 | 3,53E-05 | K562Dox | Elongation factor 2 |
| Q13200 | 3 | 182.57 | 4,02E-05 | K562 | 26S proteasome non-ATPase regulatory subunit 2 |
| P49321 | 10 | 621.17 | 4,15E-05 | K562 | Nuclear autoantigenic sperm protein |
| P02792 | 3 | 153.53 | 5,15E-05 | K562 | Ferritin light chain |
| Q09666 | 4 | 241.99 | 5,80E-05 | K562Dox | Neuroblast differentiation-associated protein AHNAK |
| Q92945 | 11 | 679.83 | 7,93E-05 | K562Dox | Far upstream element-binding protein 2 |
| Q15019 | 2 | 94.95 | 8,59E-05 | K562 | Septin-2 |
| Q16698 | 2 | 163.1 | 1,15E-04 | K562Dox | 2,4-dienoyl-CoA reductase, mitochondrial |
| Q7KZF4 | 2 | 122.97 | 1,19E-04 | K562 | Staphylococcal nuclease domain-containing protein 1 |
| Q969H8 | 2 | 128.77 | 1,31E-04 | K562Dox | UPF0556 protein C19orf10 |
| P27797 | 12 | 729.75 | 1,43E-04 | K562Dox | Calreticulin |
| O43175 | 5 | 343.55 | 2,09E-04 | K562Dox | D-3-phosphoglycerate dehydrogenase |
| Q14697 | 5 | 375.47 | 2,22E-04 | K562Dox | Neutral alpha-glucosidase AB |
| P02042 | 2 | 138.28 | 2,63E-04 | K562 | Hemoglobin subunit delta |
| Q13162 | 6 | 410.59 | 3,67E-04 | K562Dox | Peroxiredoxin-4 |
| Q9Y5M8 | 2 | 135.82 | 3,88E-04 | K562Dox | Signal recognition particle receptor subunit beta |
| O43852 | 4 | 262.95 | 5,64E-04 | K562Dox | Calumenin |
| P51572 | 2 | 116.83 | 5,80E-04 | K562Dox | B-cell receptor-associated protein 31 |

| | | | | | |
|---|---|---|---|---|---|
| O76070 | 2 | 112.31 | 8,97E-04 | K562Dox | Gamma-synuclein |
| P84095 | 2 | 122.1 | 1,13E-03 | K562 | Rho-related GTP-binding protein RhoG |
| P32119 | 5 | 372.45 | 1,35E-03 | K562Dox | Peroxiredoxin-2 |
| O43242 | 2 | 100.28 | 1,56E-03 | K562 | 26S proteasome non-ATPase regulatory subunit 3 |
| Q13838 | 3 | 145.46 | 1,72E-03 | K562 | Spliceosome RNA helicase DDX39B |
| P17174 | 2 | 88.59 | 2,79E-03 | K562 | Aspartate aminotransferase, cytoplasmic |
| P99999 | 3 | 230.82 | 3,20E-03 | K562 | Cytochrome c |
| P04792 | 4 | 255.67 | 3,45E-03 | K562Dox | Heat shock protein beta-1 |
| Q01581 | 5 | 349.64 | 3,58E-03 | K562Dox | Hydroxymethylglutaryl-CoA synthase, cytoplasmic |
| P49006 | 3 | 202.04 | 3,83E-03 | K562 | MARCKS-related protein |
| Q9NSI8 | 2 | 92.03 | 3,95E-03 | K562Dox | SAM domain-containing protein SAMSN-1 |
| O95816 | 2 | 93.14 | 1,09E-02 | K562 | BAG family molecular chaperone regulator 2 |
| Q6FI81 | 2 | 100.08 | 1,11E-02 | K562 | Anamorsin |
| Q9BRP8 | 2 | 135.58 | 1,11E-02 | K562Dox | Partner of Y14 and mago |
| P09972 | 7 | 550.98 | 1,29E-02 | K562 | Fructose-bisphosphate aldolase C |
| P09429 | 2 | 95.34 | 1,38E-02 | K562Dox | High mobility group protein B1 |
| P51571 | 2 | 134.15 | 1,56E-02 | K562Dox | Translocon-associated protein subunit delta |
| Q14103 | 2 | 133.1 | 1,75E-02 | K562 | Heterogeneous nuclear ribonucleoprotein D0 |
| P30613 | 2 | 94.94 | 2,71E-02 | K562 | Pyruvate kinase PKLR |

[a] Indicates if the protein were upregulated in K562 or K562Dox cells

**Table S2 | Statistically significant** DEPs identified with the progenesis software when comparing NCI-NCI-H460 cells with the NCI-H460/R cells.

| Accession | Peptides | Score | ANOVA (P) | Highest mean condition[a] | Description |
|---|---|---|---|---|---|
| Q13509 | 4 | 224.36 | 1,76E-09 | NCI-H460 | Tubulin beta-3 chain |
| P05120 | 7 | 398.27 | 1,24E-08 | NCI-H460/R | Plasminogen activator inhibitor 2 |
| P03956 | 2 | 147.55 | 3,55E-08 | NCI-H460/R | Interstitial collagenase |
| P26447 | 2 | 120.38 | 6,86E-08 | NCI-H460 | Protein S100-A4 |
| Q9Y2T3 | 4 | 294.37 | 8,20E-08 | NCI-H460/R | Guanine deaminase |
| P14923 | 3 | 147.28 | 9,27E-08 | NCI-H460 | Junction plakoglobin |
| Q04828;P51857 | 12 | 945.06 | 1,09E-07 | NCI-H460 | Aldo-keto reductase family 1 member C1 |
| Q9UHB6 | 4 | 263.19 | 2,31E-07 | NCI-H460/R | LIM domain and actin-binding protein 1 |
| P04350 | 3 | 189.21 | 3,27E-07 | NCI-H460 | Tubulin beta-4A chain |
| P42330 | 9 | 719.08 | 4,61E-07 | NCI-H460 | Aldo-keto reductase family 1 member C3 |
| O15067 | 3 | 163.69 | 6,01E-07 | NCI-H460 | Phosphoribosylformylglycinamidine synthase |
| O14745 | 2 | 115.52 | 9,36E-07 | NCI-H460 | Na(+)/H(+) exchange regulatory cofactor NHE-RF1 |
| P29966 | 5 | 351.65 | 1,02E-06 | NCI-H460/R | Myristoylated alanine-rich C-kinase substrate |
| P42704 | 8 | 429.18 | 1,15E-06 | NCI-H460 | Leucine-rich PPR motif-containing protein, mitochondrial |
| Q15942 | 5 | 289.61 | 1,20E-06 | NCI-H460/R | Zyxin |
| P11413 | 13 | 1012.11 | 1,22E-06 | NCI-H460 | Glucose-6-phosphate 1-dehydrogenase |
| Q96C19 | 2 | 139.32 | 1,34E-06 | NCI-H460 | EF-hand domain-containing protein D2 |
| P30043 | 3 | 158.45 | 1,42E-06 | NCI-H460 | Flavin reductase (NADPH) |
| O95994 | 6 | 421.56 | 2,01E-06 | NCI-H460/R | Anterior gradient protein 2 homolog |
| P18206 | 9 | 557.89 | 2,95E-06 | NCI-H460 | Vinculin |
| Q16799 | 2 | 118.69 | 3,10E-06 | NCI-H460 | Reticulon-1 |

| | | | | | |
|---|---|---|---|---|---|
| P52209 | 7 | 570.89 | 3,89E-06 | NCI-H460 | 6-phosphogluconate dehydrogenase, decarboxylating |
| P17812 | 3 | 149.08 | 3,93E-06 | NCI-H460/R | CTP synthase 1 |
| O43795 | 2 | 91.81 | 4,41E-06 | NCI-H460/R | Unconventional myosin-Ib |
| O60271 | 3 | 164.13 | 4,72E-06 | NCI-H460/R | C-Jun-amino-terminal kinase-interacting protein 4 |
| P08107;P17066 | 14 | 1029.09 | 5,69E-06 | NCI-H460/R | Heat shock 70 kDa protein 1A/1B |
| O75369 | 21 | 1372.59 | 5,73E-06 | NCI-H460 | Filamin-B |
| O95831 | 3 | 192.79 | 6,91E-06 | NCI-H460 | Apoptosis-inducing factor 1, mitochondrial |
| P08183 | 2 | 175.04 | 9,14E-06 | NCI-H460/R | Multidrug resistance protein 1 |
| Q16643 | 3 | 252.38 | 1,32E-05 | NCI-H460/R | Drebrin |
| P20810 | 2 | 130.59 | 1,43E-05 | NCI-H460 | Calpastatin |
| Q96B97 | 2 | 153.1 | 1,48E-05 | NCI-H460/R | SH3 domain-containing kinase-binding protein 1 |
| Q92598 | 4 | 219.52 | 1,62E-05 | NCI-H460/R | Heat shock protein 105 kDa |
| Q13501 | 7 | 676 | 1,65E-05 | NCI-H460/R | Sequestosome-1 |
| P30838 | 6 | 352.67 | 1,74E-05 | NCI-H460 | Aldehyde dehydrogenase, dimeric NADP-preferring |
| P52597 | 2 | 142.8 | 2,35E-05 | NCI-H460 | Heterogeneous nuclear ribonucleoprotein F |
| P11717 | 2 | 120.61 | 2,56E-05 | NCI-H460 | Cation-independent mannose-6-phosphate receptor |
| P43490 | 4 | 203.87 | 4,30E-05 | NCI-H460 | Nicotinamide phosphoribosyltransferase |
| P04181 | 2 | 148.51 | 6,00E-05 | NCI-H460 | Ornithine aminotransferase, mitochondrial |
| P29401 | 8 | 489.12 | 7,25E-05 | NCI-H460 | Transketolase |
| O75874 | 4 | 327.28 | 7,79E-05 | NCI-H460 | Isocitrate dehydrogenase [NADP] cytoplasmic |
| Q70E73 | 3 | 151.34 | 8,46E-05 | NCI-H460/R | Ras-associated and pleckstrin homology domains-containing protein 1 |
| P55795 | 3 | 183.51 | 8,62E-05 | NCI-H460 | Heterogeneous nuclear ribonucleoprotein H2 |
| Q9UBR2 | 2 | 95.6 | 1,18E-04 | NCI-H460 | Cathepsin Z |
| Q13557 | 2 | 121.31 | 1,24E-04 | NCI-H460/R | Calcium/calmodulin-dependent protein kinase type II subunit delta |
| P10620 | 2 | 105.19 | 1,33E-04 | NCI-H460/R | Microsomal glutathione S-transferase 1 |

| | | | | | |
|---|---|---|---|---|---|
| P46821 | 4 | 277.49 | 1,51E-04 | NCI-H460 | Microtubule-associated protein 1B |
| P00492 | 2 | 135.13 | 1,92E-04 | NCI-H460 | Hypoxanthine-guanine phosphoribosyltransferase |
| Q96HC4 | 4 | 285.33 | 1,99E-04 | NCI-H460 | PDZ and LIM domain protein 5 |
| Q8TAT6 | 2 | 88.72 | 2,07E-04 | NCI-H460/R | Nuclear protein localization protein 4 homolog |
| Q9Y2B0 | 2 | 137.58 | 2,36E-04 | NCI-H460 | Protein canopy homolog 2 |
| Q92804 | 2 | 156.39 | 3,07E-04 | NCI-H460 | TATA-binding protein-associated factor 2N |
| P12277 | 3 | 178.13 | 3,26E-04 | NCI-H460 | Creatine kinase B-type |
| P45880 | 3 | 215.06 | 4,38E-04 | NCI-H460 | Voltage-dependent anion-selective channel protein 2 |
| P37837 | 2 | 116.98 | 4,64E-04 | NCI-H460/R | Transaldolase |
| P13797;Q14651 | 3 | 184.99 | 4,83E-04 | NCI-H460 | Plastin-3 |
| P21291 | 4 | 320.55 | 5,15E-04 | NCI-H460 | Cysteine and glycine-rich protein 1 |
| O43175 | 5 | 345.57 | 7,53E-04 | NCI-H460 | D-3-phosphoglycerate dehydrogenase |
| P46940 | 7 | 407.01 | 2,20E-03 | NCI-H460/R | Ras GTPase-activating-like protein IQGAP1 |
| Q99798 | 2 | 132.35 | 2,38E-03 | NCI-H460 | Aconitate hydratase, mitochondrial |
| Q9ULV4 | 2 | 105.68 | 4,90E-03 | NCI-H460/R | Coronin-1C |
| P78527 | 2 | 142.87 | 9,44E-03 | NCI-H460/R | DNA-dependent protein kinase catalytic subunit |
| P22392 | 6 | 298.01 | 1,02E-02 | NCI-H460/R | Nucleoside diphosphate kinase B |
| P31689 | 2 | 100.79 | 1,81E-02 | NCI-H460/R | DnaJ homolog subfamily A member 1 |
| P32119 | 2 | 133.35 | 1,83E-02 | NCI-H460 | Peroxiredoxin-2 |
| P05141 | 4 | 265.29 | 2,35E-02 | NCI-H460 | ADP/ATP translocase 2 |
| P31949 | 3 | 185.3 | 2,80E-02 | NCI-H460 | Protein S100-A11 |

[a] Indicates if the protein were upregulated in NCI-NCI-H460 or NCI-NCI-H460/R cells

**Table S3 |** Intensity and Amount of the Metabolites associated with the methionine/ S-adenosylmethionine pathway.

| | Metabolite description | drug-sensitive cells | MDR cells |
|---|---|---|---|
| **K562 *vs.* K562Dox** | Methionine * | 246.2 ± 3.0 | 378.2 ± 3.6 |
| | 5´-deoxy-5´-methylthioadenosine (MTA) * | 6.8 ± 0.4 | 9.8 ± 0.3 |
| | S-adenosylhomocysteine (SAH) * | 37.5 ± 0.4 | 21.5 ± 0.5 |
| | S-adenosylmethionine (SAMe) * | 261.2 ± 3.8 | 259.1 ± 8.7 |
| | Spermidine * | 1906.8 ± 25.9 | 1611.9 ± 40.8 |
| | Betaine ** | 2041.2 ± 27.43 | 2084 ± 41.96 |
| | Choline ** | 13255 ± 43.57 | 8301 ± 113.74 |
| | Decarboxylated S-adenosyl methionine (dc-SAMe) ** | 520 ± 14.08 | 738 ± 14.05 |
| | Serine ** | 4 ± 0.00 | 2 ± 0.00 |
| | Threonine ** | 222 ± 3.51 | 220 ± 8.37 |
| **H460 vs. NCI-H460/R** | Methionine * | 473.5 ± 6.3 | 905.5 ± 20.4 |
| | 5´-deoxy-5´-methylthioadenosine (MTA) * | 2.4 ± 0.1 | 2.7 ± 0.1 |
| | S-adenosylhomocysteine (SAH) * | 143.16 ± 5.2 | 35.1 ± 0.9 |
| | S-adenosylmethionine (SAMe) * | 347.3 ± 8.0 | 343.5 ± 8.2 |
| | Spermidine * | 2627.8 ± 39.1 | 2115.9 ± 94.8 |
| | Betaine ** | 897 ± 22.89 | 1531 ± 34.79 |
| | Choline ** | 650 ± 67.41 | 3859 ± 71.03 |
| | Decarboxylated S-adenosyl methionine (dc-SAMe) ** | 44 ± 1.00 | 22 ± 0.86 |
| | Serine ** | 10 ± 0.37 | 11 ± 0.40 |
| | Threonine ** | 305 ± 7.89 | 302 ± 8.10 |

*Amount (pmol) per 1e6 cell

**Intensity (peak height)/1e6cell

**Figure S1**

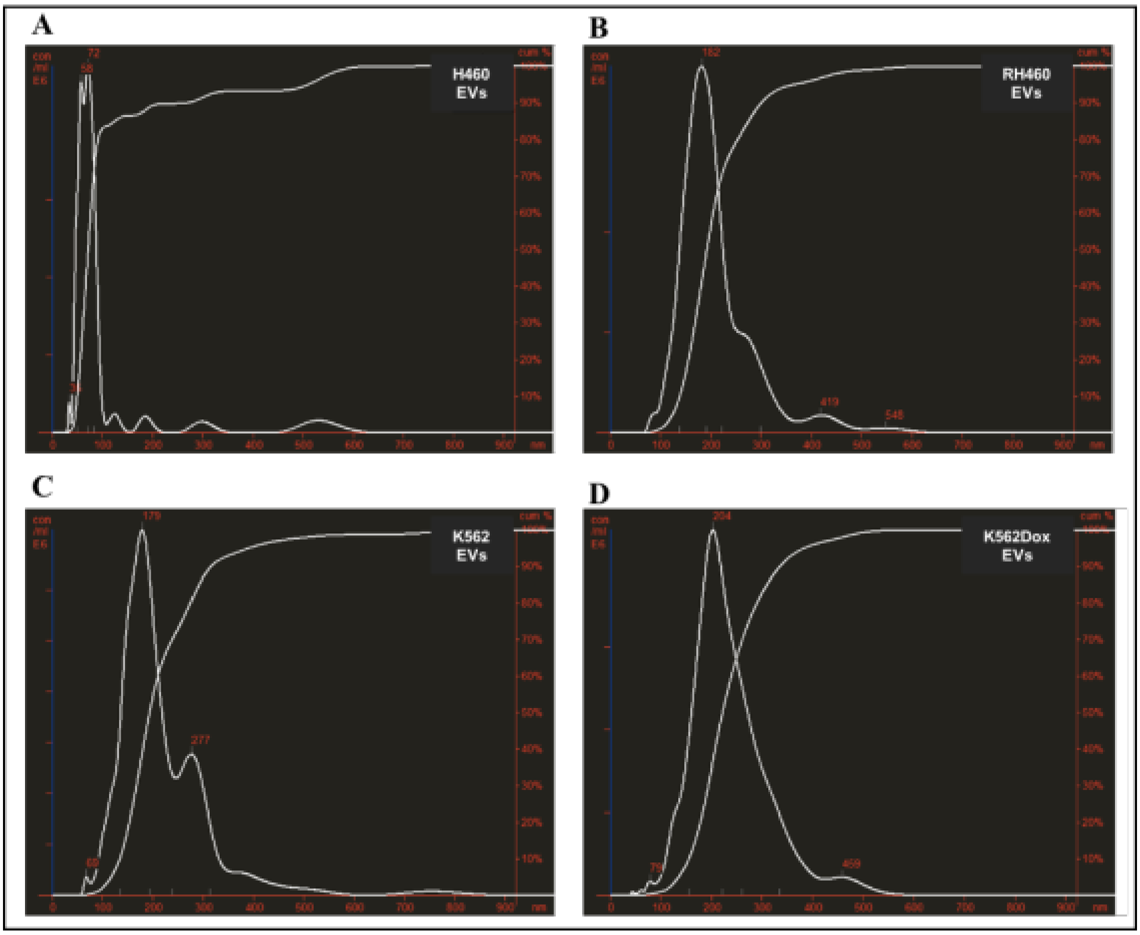


**Figure S1** – Analysis of the size distribution of the EVs released from the two pairs of drug-sensitive and MDR counterpart cell lines, obtained with the Nanosight nanoparticle tracking system. **A** – EVs isolated from NCI-H460 cells. **B** – EVs isolated from NCI-H460/R cells. **C** – EVs isolated from K562 cells. **D -** EVs isolated from K562Dox cells.
